# The Relationships between Leptin, Genotype, and Chinese Medicine Body Constitution for Obesity

**DOI:** 10.1155/2021/5510552

**Published:** 2021-05-07

**Authors:** Hsiang-I. Hou, Hsing-Yu Chen, Jang-Jih Lu, Shih-Cheng Chang, Hsueh-Yu Li, Kun-Hao Jiang, Jiun-Liang Chen

**Affiliations:** ^1^Division of Chinese Internal Medicine, Center for Traditional Chinese Medicine, Chang Gung Memorial Hospital, Taoyuan, Taiwan; ^2^Graduate Institute of Clinical Medical Sciences, College of Medicine, Chang Gung University, Taoyuan, Taiwan; ^3^School of Traditional Chinese Medicine, College of Medicine, Chang Gung University, Taoyuan, Taiwan; ^4^Department of Laboratory Medicine, Chang Gung Memorial Hospital, Taoyuan, Taiwan; ^5^Department of Medical Biotechnology and Laboratory Science, College of Medicine, Chang Gung University, Taoyuan, Taiwan; ^6^Department of Medicine, College of Medicine, Chang Gung University, Taoyuan, Taiwan; ^7^Department of Otolaryngology, Chang Gung Memorial Hospital, Gueishan County, Taoyuan, Taiwan; ^8^Department of Otolaryngology-Head and Neck Surgery, Sleep Center, Chang Gung Memorial Hospital, Taoyuan, Taiwan

## Abstract

**Methods:**

The adults with body mass index (BMI) more than 27 kg/m^2^ were enrolled in the study. General personal information, physical condition, TCMBC, biochemical, and SNPs were collected for eligible subjects. The body constitution questionnaire (BCQ) was used to evaluate the relationships between TCMBC tendency, biochemical values, and obesity-related SNPs.

**Results:**

Obesity patients tended to have a yin deficiency constitution (YinDC) (*n* = 33, 66.0%); however, TCMBC in combination is not uncommon (30 subjects with more than two TCMBC in combination). For biochemical profiles, leptin was higher among patients with yang deficiency constitution (YangDC) (YangDC versus non-YangDC: 29.7 ± 24.8 versus 15.9 ± 9.9, *P*=0.020) and YinDC (YinDC versus non-YinDC: 28.8 ± 23.5 versus 14.4 ± 9.6, *P*=0.020). The leptin level was highest among YangDC subjects. Higher leptin was found among subjects with three-combined TCMBC than balanced TCMBC subjects who were not inclined to any of three TCMBC. For obesity-related SNPs, the adrenergic receptor beta-3 (ADRB3) gene tended to be high expression among YangDC (YangDC versus non-YangDC: 89.7% versus 71.4%, *P*=0.091) and uncoupling protein 1 (UCP1) tended to be high expression among phlegm-stasis constitution (PSC) (PSC versus non-PSC: 37.9% versus 9.5%, *P*=0.052).

**Conclusions:**

The relationships between TCMBC, leptin, and SNPs present alternative viewpoints about TCMBC and could be used as a guide to treat obese patients.

## 1. Introduction

Obesity is a complex, chronic medical condition with a significant negative impact on human health [1]. Since the 21st century, the global obesity population has snowballed. Men's prevalence rate has risen from 4.8% to 9.8%, women have risen from 7.9% to 13.8%, and the global prevalence rate is 34.3% [2]. Obesity currently affects 78.6 million people (33%) in the United States and is expected to increase to over 50% of the population by 2030 [3]. In Taiwan, the prevalence of overweight is 30.5% for men and 21.3% for women, and the prevalence of obesity is 19.2% for men and 13.4% for women [4]. Obesity has become a public health burden with a significant and profound impact on morbidity, mortality, and healthcare costs [1]. Patients with obesity are at increased risk of morbidity from dyslipidemia, type 2 diabetes, hypertension, coronary heart disease, stroke, gallbladder disease, respiratory problems, sleep apnea, osteoarthritis, and some cancers [5].

There are many reasons for obesity, which can be divided into congenital and acquired. In addition to the acquired causes such as dietary habits, physical activities, and living habits, the heredity of the genetic constitution is also valued by scholars [6]. The heritability of body mass index (BMI) has been estimated to be 40–70%, and genome-wide association studies for adiposity traits have identified more than 300 single nucleotide polymorphisms (SNPs) [7, 8]. For example, the adrenergic receptor beta-2 (ADRB2), adrenergic receptor beta-3 (ADRB3), guanine nucleotide-binding protein (GNB3), uncoupling protein 1 (UCP1), and fat mass- and obesity-associated (FTO) genes are all reported to have associations with obesity. ADRB2 rs1042714 SNP may contribute to the risk of obesity and predict obesity-related metabolic traits such as BMI, triglyceride, and systolic blood pressure in Taiwanese subjects [9]. Trp64Arg (rs4994) polymorphism in the ADRB3 gene was significantly higher in overweight and obese subjects than normal weight subjects [10]. Studies regarding the role of GNB3 rs5443 polymorphism in the development of obesity and its related comorbidities are contradictory [11]. The rs1800592 of the UCP1 gene is associated with obesity in general and in the moderate obese group in particular. The associated UCP1 polymorphisms in the moderate obese group may regulate the impaired energy metabolism, which plays a significant role in the initial stages of obesity [12]. The FTO gene is highly expressed in the hypothalamus, visceral fat, and liver. It contributes to an inflammatory state, food intake, and appetite [13, 14]. A study on impaired glucose regulation patients found that leptin serum levels increased in subjects with the yang deficiency constitution and phlegm-stasis constitution, but not obese patients [15].

For traditional Chinese medicine (TCM) doctors, the constitution is one of the most important clinical references to treating diseases. The constitution is broadly defined as the fundamental components that constitute a human being and includes the total expression of physiological, psychological, and pathological traits that characterize a person's health [16]. It is a relatively stable characteristic of an organism and is affected by both nature and nurture. Traditional Chinese medicine body constitution (TCMBC) studies the overall physical condition affected by genetic and acquired factors. Body composition lays the foundation for the diagnosis, prevention, and treatment of diseases. Different constitution types make individuals susceptible to different diseases. Examining the individual's unique body constitution can promote effective health management and greatly benefit personalized medicine [16]. However, the relations between TCMBC, SNPs, and obesity-related biochemical profiles are less studied.

The present study aimed to investigate the association between TCMBC and obesity-related polymorphisms in obese patients. Our results could help to identify abnormal constitutions with a high risk for obesity and help clinicians focus on preventive strategies more effectively on these patients.

## 2. Materials and Methods

### 2.1. The Study Protocol and Enrollment

We prospectively conducted this study from January 2018 to December 2019 at the Department of Traditional Chinese Medicine of Chang Gung Memorial Hospital (Taoyuan and Taipei, Taiwan). The age, sex, BMI, waist-hip ratio, waist-height ratio, lifestyles, obesity-related genotyping and biochemical profiles, and comorbidities were collected, and TCMBC was measured. This study was approved by the Institutional Review Board (IRB) of Chang Gung Memorial Foundation (IRB no.: 201701723B0). All the study participants gave informed consent and signed a therapeutic partnership agreement. The inclusion criteria were the following: (1) BMI ≥ 27 kg/m^2^; (2) age ≥ 20 years; (3) no recognition disabilities; and (4) willing to sign informed consent and tolerance to blood sampling and questionnaire filling. The exclusion criteria were the following: (1) pregnant women or planning to conceive; (2) a history of endocrine disorders other than diabetes mellitus; (3) history of psychiatric disease and not compliance to blood sampling or questionnaire filling; (4) impaired activity of daily living; and (5) record of other ongoing clinical trials.

### 2.2. Body Constitution Questionnaire for TCMBC Assessment

Professor Yi-Chang Su kindly approved the use of the body constitution questionnaire (BCQ), Ph.D., School of Chinese Medicine, Chinese Medical University (Taichung, Taiwan), and BCQ has been widely used for TCM constitution assessments [17, 18]. All participants' TCMBC were assessed using BCQ by a TCM doctor, and the tendency to three types of TCMBC was calculated at the end of this study. The questionnaire consists of 44 items of a 5-point Likert-type response scale (from 1 (never happened) to 5 (always happens)) with a total score ranging from 44 to 220. BCQ was designed to detect the TCMBC tendency: Yang deficiency constitution (YangDC), yin deficiency constitution (YinDC), and phlegm-stasis constitution (PSC) separately. For YangDC (total score ranging from 19 to 95, 19 items), a score exceeding 31 indicated YangDC [19]; for YinDC (total score ranging from 19 to 95, 19 items), a score exceeding 30 indicated YinDC [20]; and for PSC (total score ranging from 16 to 80, 16 items), a score exceeding 27 indicated PSC [21]. A higher score implies a more significant deviation of the body constitution. Although these three TCMBC could be seen as three different, some items belonging to these three scales overlapped each other, so the body constitution classification may also be overlapped. Subjects who did not reach the threshold scores of all three imbalanced body constitutions were regarded as a balanced constitution where the lower the score, the healthier the subject. In previous studies, Cronbach's confidence *α* of each constitution's subscale ranged from 0.55 to 0.88, with the intraclass correlation coefficients exceeding 0.7 [17, 18, 21].

### 2.3. Determination of Clinical and Biochemical Profiles

The following variables were recorded for each obesity patient: gender, age, BMI, fasting sugar, hemoglobin A1C (HbA1c), estimated average glucose(eAG), high-density lipoprotein-cholesterol (HDL-c), very low-density lipoprotein (VLDL), low-density lipoprotein-cholesterol (LDL-c), total cholesterol, triglyceride (TG) leptin, insulin, and adiponectin. The homeostatic model assessment for insulin resistance (HOMA-IR) was used to represent insulin resistance.

### 2.4. Determination of SNP Selection and Genotyping

The ADRB2 gene is a vital lipolysis receptor on human fat cells. Arg16Gly (rs1042713) polymorphism of ADRB2 was significantly associated with obesity in female adolescents in Taiwan, and Gln27Glu (rs1042714) polymorphism of ADRB2 may contribute to the risk of obesity and predict obesity-related metabolic traits such as BMI, triglyceride, and systolic blood pressure in Taiwanese subjects [9, 22]. In the ADRB2 gene, Gln27Glu polymorphism is widespread in obesity and associates with increased body fat and enlarged fat cells, and Arg16Gly polymorphism is above all associated with improved adipocyte ADRB2 function [23]. Women with the Gln27Glu polymorphism in the ADRB2 gene on a high intake of carbohydrates (>50% of daily calories intake) increase the risk of obesity [24]. Those with the ADRB2 gene are called starch obesity. The ADRB3 gene regulates the lipolysis of adipose tissue. ADRB3 polymorphism might be a possible determinant of insulin resistance in Taiwanese women [25]. Middle-aged adult Asians with the ADRB3 rs4994 minor alleles are at increased risk of type 2 diabetes [26]. For people with this genotype, the lipolysis of visceral adipose tissue is reduced, which leads to higher visceral fat content, and the weight and BMI value are easily increased [27]. Those with the ADRB3 gene are called visceral obesity. The GNB3 gene slows down cell fat metabolism, causing cells to accumulate fat quickly and cause obesity [28]. The GNB3 rs5443 polymorphism is significantly associated with greater visceral fat and higher serum lipids in Korean obese women [28]. GNB3 rs5443 SNP may predict higher obesity-related metabolic traits such as triglyceride and total cholesterol in nonobese Taiwanese subjects [29]. People with the GNB3 gene are called metabolism obesity. The UCP1 gene acts on the inner mitochondrial membrane of brown fat cells and can convert energy to generate heat [30]. A Japanese study showed that UCP1 showed significant associations with visceral fat area [31]. When carrying the UCP1 gene, BMI, waist circumference, and waist-to-hip ratio values are higher than normal genes, so fat is easy to accumulate in the hips and thighs and cause lower body obesity, also known as stubborn obesity [32]. The FTO gene affects the pathway of fat cells to produce heat, so that the fat type is transformed into white fat cells, and the fat storage is increased [33]. FTO rs9939609 SNP may be linked with the risk of obesity in Taiwanese subjects [34]. The FTO gene can regulate the secretion of ghrelin and affect appetite [35]. People with the FTO gene will significantly increase their food intake, increasing the risk of rising BMI, as known as appetite obesity.

### 2.5. Physical Examination

All subjects also had physical examination analyses in this study. Bodyweight and height were measured with subjects dressed in light clothing without socks and shoes. Weight was measured (in kilograms to one decimal place) with a digital balance scale. Height was measured (in meters to one decimal place) with an electronic height measuring instrument. Body mass index (BMI) was calculated in the way that bodyweight was divided by body height squared. Waist circumference (WC) and hip circumference (HC) were measured to the nearest 0.1 cm using a standard tape. Waist circumference (WC) was measured between the lowest rib and the superior border of the iliac crest. Hip circumference (HC) was measured as the maximum circumference around the buttocks. Systolic and diastolic blood pressure was measured in the seated position with and an appropriately sized cuff on the left arm.

### 2.6. Sample Collection, SNP Selection, and Genotyping

In this study, genomic DNA was extracted from 3 mL peripheral blood samples of 50 individuals using the QIAamp DNA Blood Mini Kit according to the protocol recommended by the manufacturer (Qiagen, Valencia, CA, USA). The quantity and quality were determined using a NanoDrop (Thermo Fisher Scientific, Waltham, MA, USA). The obesity-related loci selected in this study were associated with BMI or energy intake in previously published genome-wide and candidate gene association studies (Table 1). All six obesity-related loci were genotyped in our 60 cases using the Sequenom MassARRAY platform and the standard protocol recommended by the manufacturer (Sequenom, San Diego, CA, USA). DNA samples were genotyped using the Sequenom MassARRAY platform (Agena Bioscience, San Diego, CA, USA). Primers and multiplex reactions were designed using the Agena Bioscience Assay Designer software package (v.4.0). Data management and analysis were performed using Agena Bioscience Type 4.0 software.

### 2.7. Statistical Analysis

Demographic features, including physical and biochemical profiles, were presented either by the mean and standard deviation for continuous covariates or percentage for categorical data. To compare the differences within three different TCMBC groups, Student's *t*-test was used for continuous variables, and *χ*^2^ statistics were used for categorical data. All statistics were calculated by STATA (StataCorp. 2019. Stata Statistical Software: Release 16. College Station, TX : StataCorp LLC.), and the statistics ≤0.05 were regarded as significant results.

## 3. Results

### 3.1. Subjects' Demographic Characteristics

A total of 50 obese subjects were assessed for eligibility from January 1, 2018, to December 31, 2019. After a detailed explanation of the study protocol and a full period of consideration, all patients provided informed consent. Patient characteristics, such as demographic features, TCMBC, and comorbidities data, are presented in Table 2. The study group included 13 (26.0%) males and 37 (74.0%) females with a median age of 37.0 years and an average BMI of 31.1 kg/m^2^. Obesity patients tended to have YinDC (*n* = 33, 66.0%), followed by YangDC (*n* = 29, 58.0%) and PSC (*n* = 29, 58.0%). Moreover, combined TCMBC was quite commonly seen in enrolled patients. More than half of enrolled subjects have combined TCMBC (44% of subjects had three-combined TCMBC, and 16% had two-combined TCMBC). Eleven subjects (22% of all enrolled subjects) had balanced TCMBC. The most common comorbidities of patients in this experiment were allergic rhinitis (*n* = 10, 20%) and hypertension (*n* = 8, 16%).

### 3.2. The Differences in Demographic Features among 3 TCMBC Groups

In Table 3, all demographic features and comorbidities were similar among three TCMBC groups, except gender. We found that there are differences between YangDC (female versus male: 89.7% versus 10.3%, *P*=0.003) and YinDC (female versus male: 87.9% versus 12.1%, *P*=0.002) in gender. In terms of food preferences, there is a statistical difference in the intake of icy food (PSC versus non-PSC: 34.5% versus 9.5%, *P*=0.041) and deep-fried food (PSC versus non-PSC: 31.0% versus 4.8%, *P*=0.022) in PSC. YinDC has a statistical difference in alcohol consumption (YinDC versus non-YinDC: 39.4% versus 70.6%, *P*=0.037) and diabetes mellitus (YinDC versus non-YinDC: 0.0% versus 11.8%, *P*=0.044).

### 3.3. Leptin Associated with Different TCMBC Groups

In the biochemical test, as given in Table 4, leptin was found to have statistically significant differences in the types of constitutions of YangDC (YangDC versus non-YangDC: 29.7 ± 24.8 ng/mL versus 15.9 ± 9.9 ng/mL, *P*=0.020) and YinDC (YinDC versus non-YinDC: 28.8 ± 23.5 ng/mL versus 14.4 ± 9.6 ng/mL, *P*=0.020), and we found YangDC (29.7 ± 24.8 ng/mL) has a higher leptin value than PSC (25.9 ± 21.4 ng/mL). Insulin has a statistically different trend in YinDC (YinDC versus non-YinDC: 13.0 ± 5.3 uU/mL versus 10.4 ± 4.4 uU/mL, *P*=0.088). On the other hand, blood sugar levels, lipid profiles, insulin resistance, and adiponectin were similar among different TCMBC groups.

### 3.4. The Associations between Obesity-Related Genotyping and TCMBC

Table 5 summarizes the genotyping results, and we found YangDC tended to associate with a higher mutation rate of the ADRB3 gene (proportion of high mutation rate 89.7% for YangDC and 71.4% for non-YangDC obese subjects, *P*=0.091). PSC tended to associate with the high mutation rate of the UCP1 gene (proportion of high mutation rate 37.9% for PSC and 9.5% for non-PSC obese subjects, *P*=0.052). Otherwise, FTO, GNB3, and ADRB2 had no differences among the three TCMBC groups.

### 3.5. The Comparison between Three-Combined TCMBC and Balanced Constitution

Tables 6 and 7 summarize all the study parameters and compare the different features between the three-combined and the balanced TCMBC. Overall, the tendency was similar to the findings in the comparisons within the three TCMBC. Among demographic features, subjects with three-combined TCMBC tended to be female, preferred deep-fried food, and had a diabetes mellitus history. Leptin level was markedly higher for 3-combined TCMBC subjects. For genomic profile, there were no differences between 3-combined and balanced TCMBC subjects.

## 4. Discussion

Our study found that leptin was related to YangDC and YinDC, and there was a trend between ADRB3 polymorphism rs4994 and YangDC, while there was also a similar correlation between UCP1 polymorphism rs1800592 and PSC. To the best of our knowledge, this is the first study addressing the correlations between constitution types and polymorphisms, which might help ameliorate the advances of precision TCM. TCMBC could indicate individuals' overall health status, and an unbalanced body constitution often represents deteriorated health status, a condition that Western medicine will diagnose as no disease. With objective biochemical and genomic profiles, TCMBC may help classify the obesity population in more detail as part of personalized medicine.

Most interestingly, we found that the leptin level was higher among YangDC and YinDC when compared to non-YangDC and non-YinDC subjects, respectively. The overall values of age, BMI, and high leptin were similar to other studies about the obesity population, and these facts proved the need for other classification systems. A study on the sub-Saharan African population showed that patients were about 50–55 years old, for males, the average value of BMI was 25.3 and leptin was 4.8, and for females, the average value of BMI was 28 and leptin was 18.5 [36]. Another German study showed that the included patients were 33–36 years old, for males, the average value of BMI was 26.0 and leptin was 6.5, and for females, the average value of BMI was 26.3 and leptin was 20.5 [37]. For TCMBC, the leptin serum level was higher with YangDC and PSC among the impaired glucose regulation patients [15]. Although obesity may be associated with impaired glucose regulation, the constitution seemed to be changing with different physical conditions. Besides, we found that combined TCMBC was common among obese subjects, and the tendency of higher leptin was found among three-combined TCMBC subjects. These results reflected that the mechanisms and presentations were quite complicated, and therefore, the classification of TCMBC may not be inclined to just one type in the real world.

Leptin is an adipocyte-secreted hormone that circulates primarily at levels proportional to the amount of adipose tissue, signaling long-term energy storage, and secondarily at levels modified by acute changes in caloric intake [38]. Leptin generally regulates energy homeostasis, decreases energy intake, and increases energy expenditure [38]. Obesity is characterized by elevated leptin levels or hyperleptinemia and resistance to the anorectic and bodyweight-reducing effects of leptin [39]. From the TCM's viewpoint, the body constitution means the physiological state is maintained by the dynamic combination of energy (yang) and materials (yin) in the body, just as the role of leptin in fat metabolism. The constitution theory serves as an essential foundation of clinical TCM practice and has been applied for more than 2,000 years to evaluate patients in states of subhealth, subdisease, or predisease [40]. YinDC refers to individuals' materials to perform or maintain body functions that have diminished [20]. YangDC refers to a diminishing energy level in the body's physiological functioning [19]. An imbalance between energy and material can cause both YangDC and YinDC to a considerable extent in the human body, leading to obesity, which in turn leads to higher leptin values for YangDC and YinDC subjects. Besides, obese patients with YangDC had higher leptin levels compared with the other groups. Duan et al. reported that the increased leptin level was significantly and positively correlated with BMI and waist circumference in obese women [41]. The higher leptin level increased the risk of metabolic syndrome in Taiwanese individuals [42]. YangDC is one important pathogenesis of obesity, which is a high-risk factor of metabolic syndrome and diabetes [43]. Hence, we consider that YangDC in obese patients might have a greater influence on the regulation of obesity-related hormone peptides.

Additionally, we found the most common TCMBC was YinDC, although the patient number of YinDC was close to the patient number of YangDC and PSC. Li et al. reported the significant correlations between yang deficiency and phlegm-stasis groups with overweight and obesity outcomes with a different TCMBC questionnaire [44]. A study on diagnosis in prediabetes people found that the predominant TCMBC for prediabetes people was yin deficiency [45]. A study showed that the yin deficiency type was significantly correlated with hypertension and diabetes mellitus [46]. Another study suggested that phlegm-stasis and yin deficiency patients had different hypertension [47]. The geolocation may cause the differences between these studies, since the proportion of hypertension and diabetes mellitus was both low in our study. Compared to Shang Hai, where the Li et al.' subjects' information was collected, Taiwan's latitude is lower, and the climate is warmer than Shang Hai. This fact may be the reason why the distribution of TCMBC was different from the previous study.

Moreover, we found the expression of genomic profile tended to be different among these three TCMBC. Genetic variations in the ADRB3, the *β*_3_-adrenoceptor (i.e. Try64Arg variant), are also associated with obesity. ADRB3 stimulates the mobilization of lipids from the white fat cell and increases thermogenesis in the brown fat cell. Decreased function of ADRB3 in white adipose tissue could slow lipolysis and cause the retention of lipids in fat cells [48]. YangDC implies a diminishing energy level in the body's physiological functioning and can also easily cause metabolic waste accumulation and lead to obesity [44]. UCP1 plays a vital role in metabolic and energy balance and regulation, cold- and diet-induced thermogenesis, and decreasing the production of reactive oxygen species by mitochondria, which are mechanisms associated with the pathogenesis of obesity [49, 50]. PSC refers to individuals' dynamic interaction between yin and yang that is decelerated and less efficient, and individuals may express some physical symptoms [21]. Once phlegm and stasis are formed, they would become fat accumulation and lead to obesity [44].

This study reported the relationship between TCMBC, leptin, and obesity-related SNPs. This study used biochemical parameters and genomic profiles to access TCMBC for bridging the gap between TCM theory and obesity polymorphisms. Despite its contributions, this study has several limitations. First, in our study, the patients were relatively young, and only one medical center was involved, and therefore, the generalizability may be concerned, especially the geolocation may be influential for determining TCMBC. Second, we used BCQ for assessing TCMBC, which is convenient to use and well-validated in Taiwan but has fewer constitution classifications than other studies about TCMBC [51–55]. Third, the BCQ used in this study assessed the presence or absence of three different TCMBC separately, but the three constitutions were not independent. Although it may reflect the complexity of obesity, the inference between combined TCMBC on parameters may influence the results. The constitution questionnaire to classify the subjects to a single TCMBC developed by professor Wang may be considered as well as BCQ in future studies about TCMBC classification among the obesity population [52]. Last, since this study design is cross-sectional, our study only suggested a possible association between TCMBC, leptin, and SNPs. The cause-causal relationships remain unclear, and further studies are still needed.

## 5. Conclusions

We found that the obese patients tended to have YinDC, while leptin is related to YangDC and YinDC, but not related to PSC. There is a trend towards near-statistical differences between the ADRB3 gene and YangDC, UCP1 gene, and PSC, and more research is needed to explain this relationship.

## Figures and Tables

**Table 1:** The list of obesity-related SNPs checked in this study.

Gene	Phenotype	Reference
ADRB2 rs1042713rs1042714	Starch obesity	V. Large, L. Hellstrom, S. Reynisdottir et al. “Human beta-2 adrenoceptor gene polymorphisms are highly frequent in obesity and associate with altered adipocyte beta-2 adrenoceptor function,” J Clin Invest, vol. 100, no. 12, pp. 3005–3013. J.A. Martínez, M.S. Corbalán, A. Sánchez-Villegas, L. Forga, A. Marti, and M.A. Martínez-González, “Obesity risk is associated with carbohydrate intake in women carrying the Gln27Glu beta-2 adrenoceptor polymorphism,” J Nutr, vol. 133, no. 8, pp. 2549–2554.

ADRB3 rs4994	Visceral obesity	H. Kim-Motoyama, K. Yasuda, T. Yamaguchi et al., “A mutation of the beta-3 adrenergic receptor is associated with visceral obesity but decreased serum triglyceride,” Diabetologia, vol. 40, no. 4, pp. 469–472.

GNB3 rs5443	Metabolism obesity	K.D. Ko, K.K. Kim, H.S. Suh, and I.C. Hwang, “Associations between the GNB3 C825 T polymorphism and obesity-related metabolic risk factors in Korean obese women,” J Endocrinol Invest, vol. 37, no. 11, pp. 1117–1120.

UCP1 rs1800592	Stubborn obesity	R.A. Busiello, S. Savarese, and A. Lombardi, “Mitochondrial uncoupling proteins and energy metabolism,” Front Physiol, vol. 6, p. 36. M. Dhall, M.M. Chaturvedi, U. Rai, and S. Kapoor, “Sex-dependent effects of the UCP1 -3826 A/G polymorphism on obesity and blood pressure,” Ethn Dis, vol. 22, no. 2, pp. 181–184.

FTO rs9939609	Energy intake	E. Karra, O.G. O'Daly, A.I. Choudhury et al. “A link between FTO, ghrelin, and impaired brain food-cue responsivity,” J Clin Invest, vol. 123, no. 8, pp. 3539–3551.
	BMI	M. Claussnitzer, S.N. Dankel, K.H. Kim et al., “FTO obesity variant circuitry and adipocyte browning in Humans,” N Engl J Med, vol. 373, no. 10, pp. 895–907.

**Table 2:** Baseline characteristics of enrolled obese patients (from January 1, 2018, to December 31, 2019, *n* = 50).

Parameters	Subject number (%)
Demographic features	
Gender	
Female	37 (74.0)
Male	13 (26.0)
Age (years), mean (SD)	37.0 (9.6)
BMI, mean (SD)	31.1 (3.2)
Waist-hip ratio (WHR), mean (SD)	0.9 (0.1)
Waist-height ratio (WheR), mean (SD)	0.6 (0.0)
Appetite	
Poor	2 (4.0)
Fair	43 (86.0)
Good	5 (10.0)
Food preference	
Icy food	12 (24.0)
Deep-fried food	10 (20.0)
Smoking	3 (6.0)
Alcohol consumption	25 (50.0)
Betel nuts chewing	0 (0)
Exercise ≥30 mins/week	40 (80.0)
Comorbidities	
Hyperlipidemia	2 (4.0)
Hypertension	8 (16.0)
Diabetes mellitus	2 (4.0)
Stroke	0 (0)
Myocardial infarction	0 (0)
Allergic rhinitis	10 (20.0)
Polycystic ovarian syndrome	1 (2.0)
The tendency of TCM body constitution (TCMBC)	
Yang deficiency constitution (YangDC)	29 (58.0)
Yin deficiency constitution (YinDC)	33 (66.0)
Phlegm-stasis constitution (PSC)	29 (58.0)
TCMBC in combinations	
0	11 (22.0)
1	9 (18.0)
2	8 (16.0)
3	22 (44.0)

**Table 3:** Comparisons of demographic features and comorbidities with three traditional Chinese medicine body constitutions (TCMBC).

Parameters	Yang deficiency (YangDC)			Yin deficiency (YinDC)			Phlegm-stasis (PSC)		
	(+) *n* = 29	(−) *n* = 21	*P*	(+) *n* = 33	(−) *n* = 17	*P*	(+) *n* = 29	(−) *n* = 21	*P*
Demographic features									
Gender			0.003			0.002			0.097
Female	26 (89.7%)	11 (52.4%)		29 (87.9%)	8 (47.1%)		24 (82.8%)	13 (61.9%)	
Male	3 (10.3%)	10 (47.6%)		4 (12.1%)	9 (52.9%)		5 (17.2%)	8 (38.1%)	
Age (years), mean (SD)	37.7 (10.3)	36.0 (8.7)	0.54	37.9 (9.8)	35.2 (9.2)	0.34	37.1 (10.3)	36.8 (8.8)	0.89
BMI, mean (SD)	30.8 (3.2)	31.6 (3.2)	0.42	31.1 (3.1)	31.2 (3.5)	0.93	30.8 (3.2)	31.6 (3.3)	0.43
Waist-hip ratio (WHR), mean (SD)	0.9 (0.1)	0.9 (0.0)	0.20	0.9 (0.1)	0.9 (0.0)	0.19	0.9(0.1)	0.9 (0.0)	0.56
Waist-height ratio (WheR), mean (SD)	0.6 (0.1)	0.6 (0.0)	0.67	0.6 (0.0)	0.6 (0.0)	0.35	0.6 (0.1)	0.6 (0.0)	0.24
Appetite			0.10			0.57			0.35
Poor	2 (6.9%)	0 (0.0%)		2 (6.1%)	0 (0.0%)		2 (6.9%)	0 (0.0%)	
Fair	26 (89.7%)	17 (81.0%)		28 (84.8%)	15 (88.2%)		25 (86.2%)	18 (85.7%)	
Good	1 (3.4%)	4 (19.0%)		3 (9.1%)	2 (11.8%)		2 (6.9%)	3 (14.3%)	
Food preference									
Icy food	9 (31.0%)	3 (14.3%)	0.17	9 (27.3%)	3 (17.6%)	0.45	10 (34.5%)	2 (9.5%)	0.041
Deep-fried food	8 (27.6%)	2 (9.5%)	0.12	8 (24.2%)	2 (11.8%)	0.30	9 (31.0%)	1 (4.8%)	0.022
Smoking	2 (6.9%)	1 (4.8%)	0.75	2 (6.1%)	1 (5.9%)	0.98	2 (6.9%)	1 (4.8%)	0.75
Alcohol consumption	12 (41.4%)	13 (61.9%)	0.15	13 (39.4%)	12 (70.6%)	0.037	13 (44.8%)	12 (57.1%)	0.39
Exercise ≥30 mins/week	23 (79.3%)	17 (81.0%)	0.89	27 (81.8%)	13 (76.5%)	0.65	24 (82.8%)	16 (76.2%)	0.57
Comorbidities									
Hyperlipidemia	1 (3.4%)	1 (4.8%)	0.82	2 (6.1%)	0 (0.0%)	0.30	1 (3.4%)	1 (4.8%)	0.82
Hypertension	5 (17.2%)	3 (14.3%)	0.78	5 (15.2%)	3 (17.6%)	0.82	5 (17.2%)	3 (14.3%)	0.78
Diabetes mellitus	0 (0.0%)	2 (9.5%)	0.090	0 (0.0%)	2 (11.8%)	0.044	0 (0.0%)	2 (9.5%)	0.090
Stroke	0	0	1.00	0	0	1.00	0	0	1.00
Myocardial infarction	0	0	1.00	0	0	1.00	0	0	1.00
Allergic rhinitis	6 (20.7%)	4 (19.0%)	0.89	8 (24.2%)	2 (11.8%)	0.30	8 (27.6%)	2 (9.5%)	0.12
Polycystic ovarian syndrome	1 (3.4%)	0 (0.0%)	0.39	0	0	1.00	0 (0.0%)	1 (4.8%)	0.24

**Table 4:** Comparisons of biochemical profiles within 3 traditional Chinese medicine body constitutions (TCMBC).

Parameters	Yang deficiency (YangDC)			Yin deficiency (YinDC)			Phlegm-stasis (PSC)		
	(+) *n* = 29	(−) *n* = 21	*P*	(+) *n* = 33	(−) *n* = 17	*P*	(+) *n* = 29	(−) *n* = 21	*P*
Fasting sugar	92.7 (12.7)	99.3 (24.6)	0.22	94.1 (12.2)	98.2 (27.6)	0.47	92.9 (13.0)	99.0 (24.5)	0.25
HbA1c	6.0 (0.4)	6.0 (0.7)	0.93	6.0 (0.4)	6.0 (0.7)	0.86	6.0 (0.4)	6.0 (0.7)	0.72
eAG	126.4 (11.7)	126.0 (19.2)	0.94	125.9 (11.6)	126.8 (20.8)	0.85	126.9 (11.6)	125.4 (19.3)	0.74
HDL	48.3 (10.9)	51.0 (19.3)	0.53	47.6 (10.5)	52.9 (21.0)	0.24	46.8 (9.9)	53.1 (19.6)	0.14
VLDL	28.3 (20.2)	33.1 (19.1)	0.41	32.7 (20.5)	25.3 (17.4)	0.22	30.4 (20.3)	30.1 (19.2)	0.96
LDL	129.9 (35.0)	131.1 (36.7)	0.91	130.6 (36.4)	130.0 (34.2)	0.95	130.0 (36.1)	131.0 (35.1)	0.93
Total cholesterol	199.8 (41.5)	203.1 (36.2)	0.77	203.2 (40.1)	197.4 (37.8)	0.63	199.7 (43.2)	203.2 (33.2)	0.76
Total cholesterol/HDL	4.3 (1.2)	4.4 (0.9)	0.70	4.4 (1.1)	4.3 (1.2)	0.63	4.4 (1.2)	4.3 (1.0)	0.73
LDL/HDL	2.8 (1.0)	2.8 (0.7)	0.94	2.8 (0.8)	2.8 (1.0)	0.99	2.9 (0.9)	2.7 (0.8)	0.57
Triglyceride	146.2 (120.0)	181.7 (139.1)	0.34	175.7 (140.0)	132.9 (99.4)	0.27	156.6 (120.5)	167.5 (141.0)	0.77
Leptin	29.7 (24.8)	15.9 (9.9)	0.020	28.8 (23.5)	14.4 (9.6)	0.020	25.9 (21.4)	21.0 (20.5)	0.42
Insulin	11.9 (5.6)	12.4 (4.5)	0.73	13.0 (5.3)	10.4 (4.4)	0.088	12.3 (5.0)	11.9 (5.4)	0.79
HOMA-IR	2.8 (1.7)	3.6 (3.0)	0.21	3.1 (1.6)	3.3 (3.4)	0.82	2.9 (1.6)	3.5 (3.1)	0.35
Adiponectin	7.4 (5.0)	7.6 (4.4)	0.90	7.6 (5.1)	7.2 (4.0)	0.78	7.4 (5.2)	7.6 (4.0)	0.87

HbA1c, glycated hemoglobin; eAG, estimated average glucose; HDL, high-density lipoprotein; VLDL, very low-density lipoprotein; LDL, low-density lipoprotein; HOMA-IR, homeostatic model assessment for insulin resistance.

**Table 5:** Comparisons of genomic profiles within 3 traditional Chinese medicine body constitutions (TCMBC).

Parameters			Yang deficiency (YangDC)			Yin deficiency (YinDC)			Phlegm-stasis (PSC)		
			(+) *n* = 29	(−) *n* = 21	*P*	(+) *n* = 33	(−) *n* = 17	*P*	(+) *n* = 29	(−) *n* = 21	*P*
Obesity polymorphisms											
ADRB2 (starch)	rs1042713	Low	11 (37.9%)	5 (23.8%)	0.50	13 (39.4%)	3 (17.6%)	0.20	9 (31.0%)	7 (33.3%)	0.79
		Medium	12 (41.4%)	12 (57.1%)		13 (39.4%)	11(64.7%)		15 (51.7%)	9 (42.9%)	
		High	6 (20.7%)	4 (19.0%)		7 (21.2%)	3 (17.6%)		5 (17.2%)	5 (23.8%)	
	rs1042714	Low	27 (93.1%)	19 (90.5%)	0.74	31 (93.9%)	15 (88.2%)	0.48	27 (93.1%)	19 (90.5%)	0.74
		Medium	2 (6.9%)	2 (9.5%)		2 (6.1%)	2 (11.8%)		2 (6.9%)	2 (9.5%)	
		Low	1 (3.4%)	0 (0.0%)	0.091	1 (3.0%)	0 (0.0%)	0.15	1 (3.4%)	0 (0.0%)	0.63

ADRB3 (visceral)	rs4994	Medium	2 (6.9%)	6 (28.6%)		3 (9.1%)	5 (29.4%)		4 (13.8%)	4 (19.0%)	
		High	26 (89.7%)	15 (71.4%)		29 (87.9%)	12 (70.6%)		24 (82.8%)	17 (81.0%)	
		Low	3 (10.3%)	3 (14.3%)	0.70	3 (9.1%)	3 (17.6%)	0.46	2 (6.9%)	4 (19.0%)	0.42

GNB3 (metabolism)	rs5443	Medium	13 (44.8%)	11 (52.4%)		15 (45.5%)	9 (52.9%)		15 (51.7%)	9 (42.9%)	
		High	13 (44.8%)	7 (33.3%)		15 (45.5%)	5 (29.4%)		12 (41.4%)	8 (38.1%)	

UCP1 (stubborn)	rs1800592	Low	3 (10.3%)	5 (23.8%)	0.18	4 (12.1%)	4 (23.5%)	0.45	5 (17.2%)	3 (14.3%)	0.052
		Medium	16 (55.2%)	13 (61.9%)		19 (57.6%)	10 (58.8%)		13 (44.8%)	16 (76.2%)	
		High	10 (34.5%)	3 (14.3%)		10 (30.3%)	3 (17.6%)		11 (37.9%)	2 (9.5%)	

FTO (appetite)	rs9939609	Low	20 (69.0%)	15 (71.4%)	0.46	24 (72.7%)	11 (64.7%)	0.37	20 (69.0%)	15 (71.4%)	0.46
		Medium	7 (24.1%)	6 (28.6%)		7 (21.2%)	6 (35.3%)		7 (24.1%)	6 (28.6%)	
		High	2 (6.9%)	0 (0.0%)		2 (6.1%)	0 (0.0%)		2 (6.9%)	0 (0.0%)	

**Table 6:** Comparisons of demographic features, comorbidities, biochemical profiles, and genomic profiles within three-combined traditional Chinese medicine body constitutions (TCMBC) and balanced constitution (no inclined to any of three types) obesity enrollees.

Parameters	TCMBC		
	Three-combined (*n* = 22)	Balanced (*n* = 11)	*P*
Demographic features			
Gender			<0.001
Female	20 (90.9%)	4 (36.4%)	
Male	2 (9.1%)	7 (63.6%)	
Age (years), mean (SD)	38.4 (10.9)	35.0 (10.2)	0.40
BMI, mean (SD)	30.5 (3.1)	31.3 (3.3)	0.50
Waist-hip ratio (WHR), mean (SD)	0.9 (0.1)	0.9 (0.0)	0.27
Waist-height ratio (WheR), mean (SD)	0.6 (0.1)	0.6 (0.0)	0.47
Appetite			0.53
Poor	2 (9.1%)	0 (0.0%)	
Fair	19 (86.4%)	10 (90.9%)	
Good	1 (4.5%)	1 (9.1%)	
Food preference			
Icy food	7 (31.8%)	1 (9.1%)	0.15
Deep-fried food	6 (27.3%)	0 (0.0%)	0.056
Smoking	1 (4.5%)	0 (0.0%)	0.47
Alcohol consumption	7 (31.8%)	7 (63.6%)	0.081
Exercise ≥30 mins/week	17 (77.3%)	8 (72.7%)	0.77
Comorbidities			
Hyperlipidemia	1 (4.5%)	0 (0.0%)	0.47
Hypertension	4 (18.2%)	2 (18.2%)	1.00
Diabetes mellitus	0 (0.0%)	2 (18.2%)	0.039
Stroke	0	0	1.00
Myocardial infarction	0	0	1.00
Allergic rhinitis	6 (27.3%)	1 (9.1%)	0.23
Polycystic ovarian syndrome	0	0	1.00
Biochemical profiles			
Fasting sugar	92.4 (13.1)	101.7 (33.2)	0.25
HbA1c	6.1(0.5)	6.1 (0.9)	0.74
eAG	127.0 (13.0)	129.4 (25.7)	0.73
HDL	47.6 (10.3)	55.1 (24.6)	0.23
VLDL	31.5 (21.9)	31.6 (19.2)	0.99
LDL	126.9 (34.1)	129.9 (27.7)	0.80
Total cholesterol	199.2 (42.8)	201.6 (29.3)	0.87
Total cholesterol/HDL	4.3 (1.2)	4.4 (1.0)	0.92
LDL/HDL	2.8 (0.9)	2.8 (0.7)	0.89
Triglyceride	163.2 (132.2)	165.1 (110.7)	0.97
Leptin	27.7 (23.9)	11.3 (8.8)	0.036
Insulin	12.1 (5.3)	11.1 (4.7)	0.59
HOMA-IR	2.9 (1.7)	3.9 (4.0)	0.29
Adiponectin	7.2 (5.2)	6.5 (3.2)	0.70

HbA1c, glycated hemoglobin; eAG, estimated average glucose; HDL, high-density lipoprotein; VLDL, very low-density lipoprotein; LDL, low-density lipoprotein; HOMA-IR, homeostatic model assessment for insulin resistance.

**Table 7:** The genomic profile difference among three-combined traditional Chinese medicine body constitutions (TCMBC) and balanced constitution (not inclined to any of three types) obesity enrollees.

Obesity polymorphisms	TCMBC				*P*
ADRB2 (starch)	rs1042713	Low	8 (36.4%)	2 (18.2%)	0.33
		Medium	10 (45.5%)	8 (72.7%)	
		High	4 (18.2%)	1 (9.1%)	
	rs1042714	Low	20 (90.9%)	9 (81.8%)	0.45
		Medium	2 (9.1%)	2 (18.2%)	

ADRB3 (visceral)	rs4994	Low	1 (4.5%)	0 (0.0%)	0.14
		Medium	1 (4.5%)	3 (27.3%)	
		High	20 (90.9%)	8 (72.7%)	

GNB3 (metabolism)	rs5443	Low	1 (4.5%)	2 (18.2%)	0.34
		Medium	11 (50.0%)	6 (54.5%)	
		High	10 (45.5%)	3 (27.3%)	

UCP1 (stubborn)	rs1800592	Low	3 (13.6%)	3 (27.3%)	0.28
		Medium	9 (40.9%)	6 (54.5%)	
		High	10 (45.5%)	2 (18.2%)	

FTO (appetite)	rs9939609	Low	16 (72.7%)	8 (72.7%)	0.53
		Medium	4 (18.2%)	3 (27.3%)	
		High	2 (9.1%)	0 (0.0%)	

## Data Availability

The data used to support the findings of this study are available from the corresponding author upon request.

## Abbreviations

ADRB2: Adrenergic receptor beta-2
ADRB3: Adrenergic receptor beta-3
BCQ: Body constitution questionnaire
BMI: Body mass index
eAG: Estimated average glucose
FTO: Fat mass- and obesity-associated
GNB3: Guanine nucleotide-binding protein
HbA1c: Hemoglobin A1C
HC: Hip circumference
HDL-C: High-density lipoprotein-cholesterol
HOMA-IR: Homeostatic model assessment for insulin resistance
IRB: Institutional Review Board
LDL-C: Low-density lipoprotein-cholesterol
PSC: Phlegm-stasis constitution
SNPs: Single nucleotide polymorphisms
TCM: Traditional Chinese medicine
TCMBC: Traditional Chinese medicine body constitution
TG: Triglyceride
UCP1: Uncoupling protein 1
VLDL: Very low-density lipoprotein
WC: Waist circumference
YangDC: Yang deficiency constitution
YinDC: Yin deficiency constitution.
